# Visual display for surgical targeting: concepts and usability study

**DOI:** 10.1007/s11548-021-02355-8

**Published:** 2021-04-08

**Authors:** Milovan Regodić, Zoltán Bárdosi, Georgi Diakov, Malik Galijašević, Christian F. Freyschlag, Wolfgang Freysinger

**Affiliations:** Medical University of Innsbruck, Innsbruck, Austria

**Keywords:** Image-guided surgery, Visual guidance, Usability study, Surgical targeting, Electromagnetic tracking

## Abstract

**Purpose:**

Interactive image-guided surgery technologies enable accurate target localization while preserving critical nearby structures in many surgical interventions. Current state-of-the-art interfaces largely employ traditional anatomical cross-sectional views or augmented reality environments to present the actual spatial location of the surgical instrument in preoperatively acquired images. This work proposes an alternative, simple, minimalistic visual interface intended to assist during real-time surgical target localization.

**Methods:**

The estimated 3D pose of the interventional instruments and their positional uncertainty are intuitively presented in a visual interface with respect to the target point. A usability study with multidisciplinary participants evaluates the proposed interface projected in surgical microscope oculars against cross-sectional views. The latter was presented on a screen both stand-alone and combined with the proposed interface. The instruments were electromagnetically navigated in phantoms.

**Results:**

The usability study demonstrated that the participants were able to detect invisible targets marked in phantom imagery with significant enhancements for localization accuracy and duration time. Clinically experienced users reached the targets with shorter trajectories. The stand-alone and multi-modal versions of the proposed interface outperformed cross-sectional views-only navigation in both quantitative and qualitative evaluations.

**Conclusion:**

The results and participants’ feedback indicate potential to accurately navigate users toward the target with less distraction and workload. An ongoing study evaluates the proposed system in a preclinical setting for auditory brainstem implantation.

**Supplementary Information:**

The online version contains supplementary material available at 10.1007/s11548-021-02355-8.

## Introduction

Within the last decades, surgeons have been aided with image-guided, computer-assisted surgery (IGS) for accurate intraoperative navigation and localization of important critical structures in many interventions. Navigation is particularly useful in neurosurgery [1]. The positioning of deep brain electrodes is assisted by localizing preoperatively planned targets in the brain for the treatment of epilepsy and Parkinson’s disease while at the same time preventing damages to nearby blood vessels [1]. Similarly, in intracranial biopsy (where tissue samples are taken by a probe), the tool-tip is guided to target points inside the brain [1]. Another application of IGS is to preserve the facial nerve and cochlea during petrous bone drilling preceding the insertion of a cochlear implant in the middle-ear [2]. In radiofrequency (RF) ablation therapies—which are often used to treat tumors with large volume and/or irregular shape—alternating current high-frequency radio waves, dissipated at the tip of an electrode, are used to destroy tumor tissue while simultaneously minimizing damages to neighboring normal tissues [3]. IGS navigation is vital to repeatedly and reliably reach the designated target locations during the aforementioned medical procedures.

The real-time tracking of the patient and various surgical tools are crucial building blocks. The two most widely used technologies are optical (OT) and electromagnetic tracking (EMT). OT is very accurate, but suffers from line of sight deficiency, which may limit surgeons during interventions, especially in the head-neck area, where the operating area is small, and cluttered. Although EMT provides inferior accuracy and is prone to ferromagnetic distortions, it is complementary to OT. It includes freehand movements and flexible and miniature sensors that can be mounted on surgical instruments and endoscopes.

Another central component in IGS is patient-to-image registration which relates the preoperative imagery with the intraoperative scene. In neurosurgery and RF ablation, landmark-based methods can be used with corresponding fiducials localized in both patient and image space [4].

Although certain steps can be automatized as fiducial localization [5, 6], the computer-assisted navigation, as defined in [7], operates with low-degree of automation since it only influences decision-making process by presenting the information, while the execution is still manual by surgeon. Therefore, understanding human–computer interaction (HCI) is of the vital importance for implementing easier, useful and efficient interfaces that will lead to increased quality of clinical outcomes and improved patient safety. In fact, awareness of usability factors is required by regulations for medical devices in order to ensure intended use and prevent potential use errors [8]. Today’s most widely used HCIs are based on visual displays [2, 7, 9]. Though auditory displays are also reported [10] to help achieve blind placement within satisfactory accuracy margins and reduce workload, they are mostly considered as additional feedback to the visual assistance such as audio alarming for warnings/errors or improved depth perception. Both displays can free up additional resources and allow surgeons and staff to focus on more critical and safety related tasks during surgery, with the visual allowing better real-time 3D spatial perception [11]. Along with these benefits, in a questionnaire study [9], surgeons reported additional cognitive demands and worse (stress related) performance (e.g., time pressure) during surgery by using IGS compared to human (alone) operation—without any degree of automation. As they point out, this is due to the fact that on a basic level—which is the most common in surgical rooms—the visualization is displayed on a separate monitor, thus requiring surgeon to divert attention and look away from the surgical scene to obtain information. This potentially can not only be inefficient for surgical performance but also a cause for errors.

Albeit the surgeon’s focus on the scene could be maintained by projecting visualization in the microscope, this setup traditionally relates spatial localization of surgical instruments in cross-sectional and volumetric patient’s images [2], which poses risks to generate information overload while at the same time occupies the view of the operating field. The systems with augmented reality (AR) are increasingly popular and provide another way with direct guidance in a surgical microscope [12]. AR usually implements simple semi-transparent visual objects from preoperative images such as target location or contours of critical anatomical structures overlaid on the patient’s intraoperative anatomy. However, these systems have found only limited clinical use due to the increased complexity and uncertainty related to spatial registration between virtual and real structures, microscope tracking, depth perception and visual temporal asynchrony [12].

This work describes a novel visual guidance interface that aims to assist for simultaneous target localization during surgical procedures. Simple, instinctive, semi-transparent virtual cues guide surgeons toward the specified target point; however, in contrast to AR, without the necessity of spatial registration of the visualization instruments (such as the microscope optics and cameras). The proposed visualization can be used as a stand-alone overlay over the endoscopic or microscopic views, or as a multi-modal guide, when coupled with more conventional cross-sectional views. As a proof of concept, the usefulness of such overlays is demonstrated by localizing targets in customized phantoms using EMT navigated interventional instruments. The usability-related aspects are studied in order to evaluate user performance as well as to determine areas of improvements.

This paper is an extended version of the contribution presented at the CARS / CURAC 2020 conferences that were focused to demonstrate the software design principles and included preliminary results [13, 14].

## Methods

### Surgical navigation

For the purposes of tracking surgical instruments and performing image registration as well as presenting cross-sectional views navigation employed in the usability study (see Sect. 2.5), an IGS system was utilized. The system is developed in-house and follows modular software design principles [13].

### Visual guidance

We developed a simple interface for visual orientation using virtual cues that concurrently project the distance between the instrument tip and the designed target position.

**Fig. 1:**
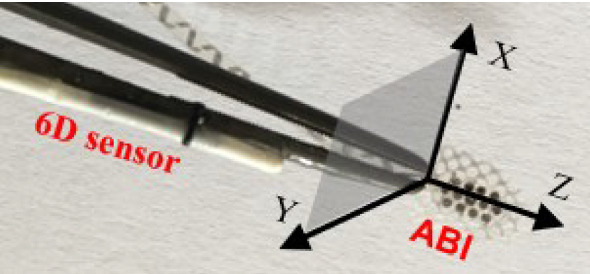
The titanium surgical forceps grasping an auditory brainstem implant electrode (ABI) and tracked with an Aurora 6D electromagnetic sensor attached on one shank of the forceps. The direction perpendicular to the instrument is XY plane termed as “lateral” and along the instrument Z distance termed as “depth”

As aforementioned, the distance $\Delta d$ is measured and represents a translation in millimeters between instrument tip $p_{tip}$ and target $p_{target}$ positions, as follows:

1 $$\begin{aligned} \Delta d=p_{{\text {tip}}}-p_{{\text {target}}}, \end{aligned}$$

where $\Delta d$, $p_{tip}$ and $p_{target}$ are 3D points in $R^3$ with components (*x*, *y*, *z*) associated with the preoperative image coordinate frame. In general, the distance is determined by 3 translation axes from the viewpoint of the observer: moving in/out, up/down and left/right. We set those components so that, for a certain orientation of the surgical instrument with respect to the image planes, the *z*-axis runs longitudinally along the direction of the instrument tip and represents movement in/out, whereas the axes *x* and *y* lie in a plane that is perpendicular to the *z* axis (representing up/down and left/right) as illustrated in Fig. 1. The first direction is termed as “depth” and the second as “lateral”. It is important to note that the image frame of reference was used only to calculate the displacements. In order to visualize them on a 2D visual plane, so that the user obtains spatial perception of a 3D tool-tip position with respect to the target in anatomy, we established another frame of reference for the following visual encoding.

**Fig. 2:**
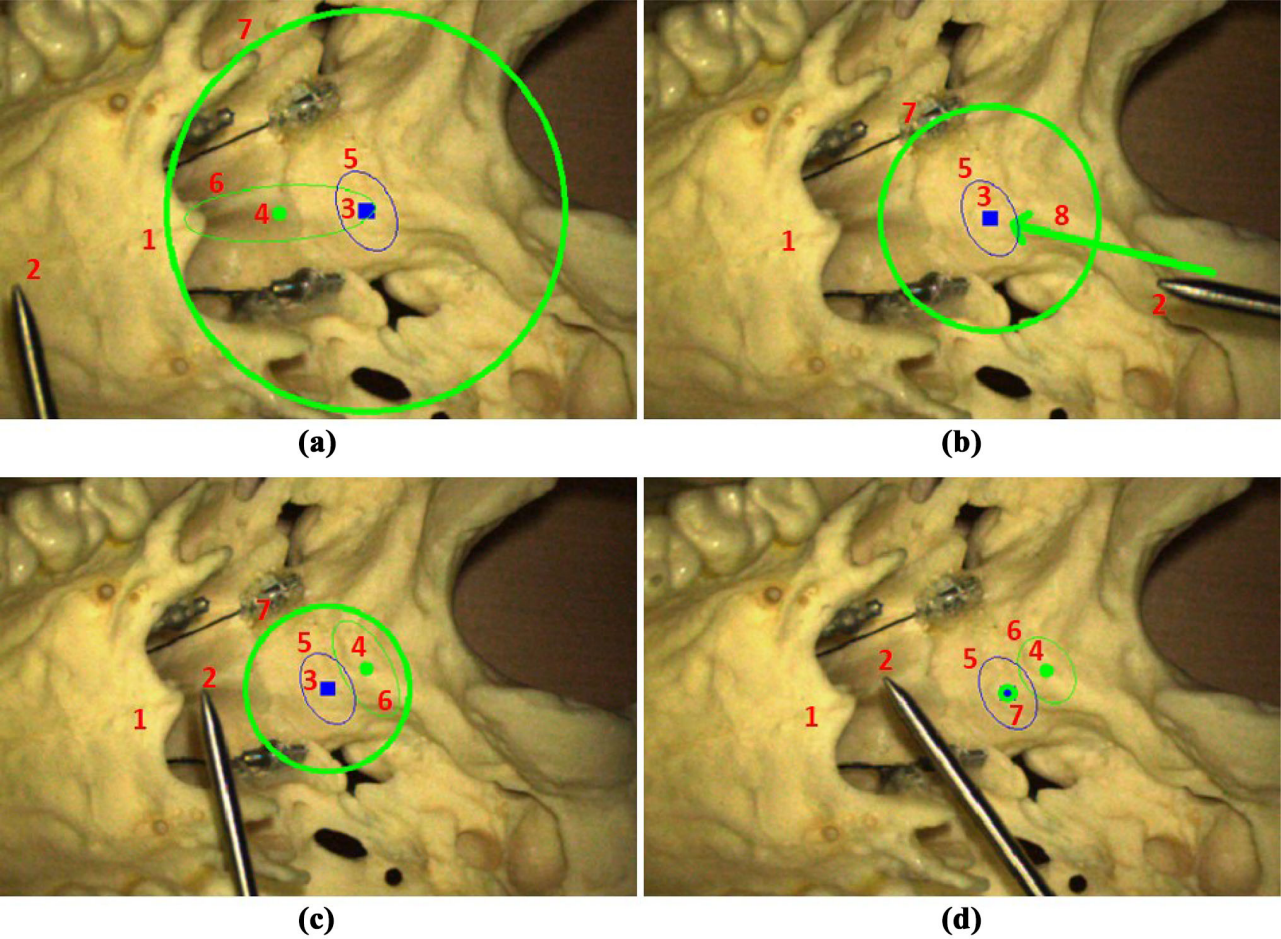
The virtual cues superimposed on the view from below on the inferior surface of the synthetic skull base in a surgical microscope Leica M500 N (Leica Microscopy Systems, Heerbrugg, Switzerland). The red-colored numbers indicate the specific objects in the images. The green-colored virtual cues encode the pointer while blue-colored the fixed target. (Images a/b/d appeared previously in [13] are used by permission)

First, we define a local coordinate system that has an origin at pixel location *T* in the visual plane such as a 2D image sampled in the microscope oculars. Then, the virtual cues superimposed onto the visual scene are positioned relative to this origin. The virtual cue representing the target is visualized as a small filled square with constant length and center exactly at origin *T* (Fig. 2 number 3). Likewise, a small filled circle with the diameter same as the target’s square length is symbolized for the current position of the tip in the lateral direction (Fig. 2 number 4). The center of this circle is located at a “moving” pixel location *L* that is calculated relative to the target location *T*:

2 $$\begin{aligned} L(\Delta d_x,\Delta d_y)= T - \frac{\begin{bmatrix} \Delta d_x&\Delta d_y \end{bmatrix}}{S_{{\text {motion}}}}, \end{aligned}$$

where $S_{motion}$ is a scaling constant. The spatial position in the lateral direction is perceived by minimizing the distance between the two virtual cues until they overlap each other.

At distances outside of the defined range $d_{max}$ where $\sqrt{{\Delta d_x}^2+{\Delta d_y}^2}>d_{max}$, an arrow is visualized instead of the small circle (Fig. 2b number 8). This arrow points from the (hidden) in-plane representation of the tip to the target visual cue with a constant length $d_{max}$ and an angle $atan2(\Delta d_x,\Delta d_y)$.

In contrast, the depth distance $\Delta d_z$, which encodes moving in/out, is projected as a “fat” circle centered at the target *T* (Fig. 2 number 7). The spatial position is perceived by adapting the radius of this circle $r_{depth}$, so that the distance between the current position of the tip and the target in the depth direction is completely minimized when $r_{depth}=0$.

At distances outside of the defined range $d_{max}$ where $|\Delta d_z|>d_{max}$, the circle diameter stays constant. This said, we formalize as follows:

3 $$\begin{aligned} r_{{\text {depth}}}(\Delta d_z)=\frac{1}{S_{{\text {motion}}}} {\left\{ \begin{array}{ll} \Delta d_z, &{} \text {if}\ |\Delta d_z| \le d_{{\text {max}}} \\ d_{{\text {max}}}, &{} \text {otherwise} \end{array}\right. } \end{aligned}$$

Beyond the target $\Delta d_z<0$, the circle blinks periodically to signalize that the position has outreached the target point.

The maximum virtual divergence of the lateral and depth cues from the target cue is bounded by a constant $d_{max}$ in order to have them always within the display field of view. The movement of cues toward or away from the target cue is invariant to the direction and measurable in millimeters through the application of a multiplicative scaling constant $S_{motion}$. The scale gives intuitive sense of physical dimensions and can be adjusted for optimum rate desired by surgeon during visualization.

Figure 2 illustrates the mechanisms formalized above for four positions of the surgical pointer in different phantom scenes sampled in a surgical microscope. The pointer (number 2) is navigated with respect to the target (number 1). The target is positioned inferiorly in the medial line at the point of articulation of two contralateral horizontal parts of the palatine bone. The pointer tip is “laterally” displaced left and right from the target in Fig. 2a–d, respectively. In the depth direction, the tip is progressively moved toward the target from a larger distance shown by a minimized depth circle in Fig. 2a–d. In Fig. 2d, the minimization circle occludes the target cue. Please note that the “depth” displacement cannot be clearly perceived due to 2D images. For instance, Fig. 2c, d encode the same lateral displacement, but at the two different depth positions.

A supplementary video clip is available for better interpretation.

### Pose estimation of surgical instruments

The pose of surgical instruments is estimated with EMT sensors attached to the instruments. In addition to a surgical probe that has a 6D sensor mounted, we also attach a 6D sensor on a titanium surgical forceps (B. Braun AG, Melsungen, Germany) which typically aids during brain operations, such as tumor removal, or by grasping implantation electrodes (Fig. 1) during auditory brainstem implantation (ABI). The 6D sensor position is pivot-calibrated at the forceps tip.

### Estimating positional uncertainty

The quality of landmark registration is directly related to fiducial identification in both image and patient. The imperfection of this step results in displacements from correct (actual) location commonly termed as fiducial localization error (FLE). The FLE directly governs both qualitative measures: fiducial registration error (FRE) and target registration error (TRE) [4]. The FRE is equal to the squared distance of corresponding fiducial pairs in image and physical space after registration. The TRE is a more meaningful measure of accuracy as it is directly estimated at a surgical point of interest [4]. Danilchenko and Fitzpatrick [15] developed generalized approach for a first-order approximation of TRE. Their algorithm is implemented here to provide a feedback for surgeons concerning positional uncertainty of the instrument localization. Following the TRE statistical model with the chi-square distribution [15], the 95% confidence region of the calculated TRE in the lateral direction is visualized as green and blue error ellipses for the current instrument tip and the target position as depicted in Fig. 2 (numbers 5 and 6).

**Fig. 3:**
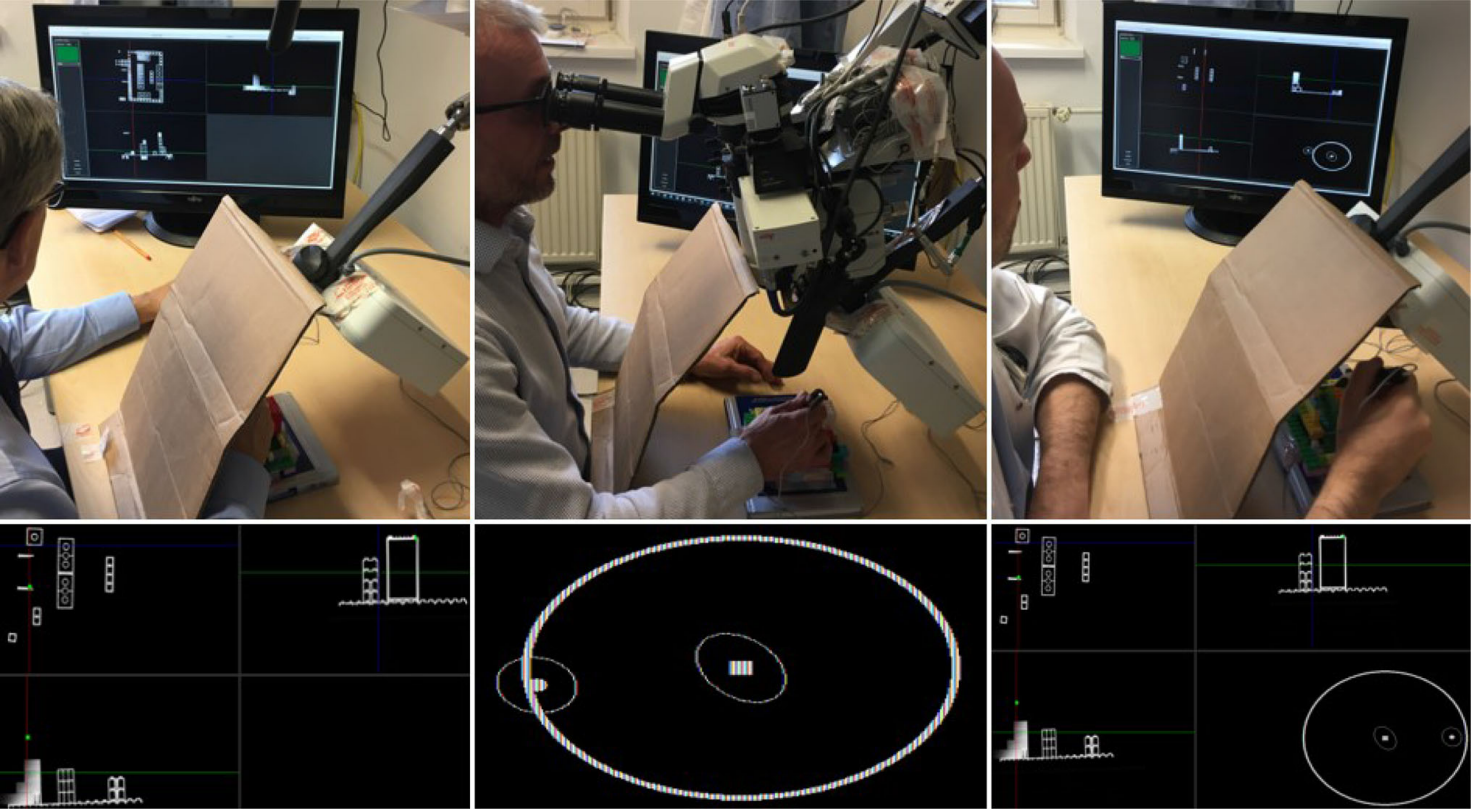
In the figures above, participants performing the experiment with a covered Lego scene using system display as shown in the figures below for: **a** IGS, **b** VG and **c** IGS+VG

### Usability study

Usability was assessed as defined by medical standard IEC62366 [8] with operating principles that demonstrate intended use and human factors: learnability—how fast user gets acquainted with the system; satisfaction—what is the overall user impression, discomfort and positive attitudes; and memorizability—how easy it is to remember system details.

**Fig. 4:**
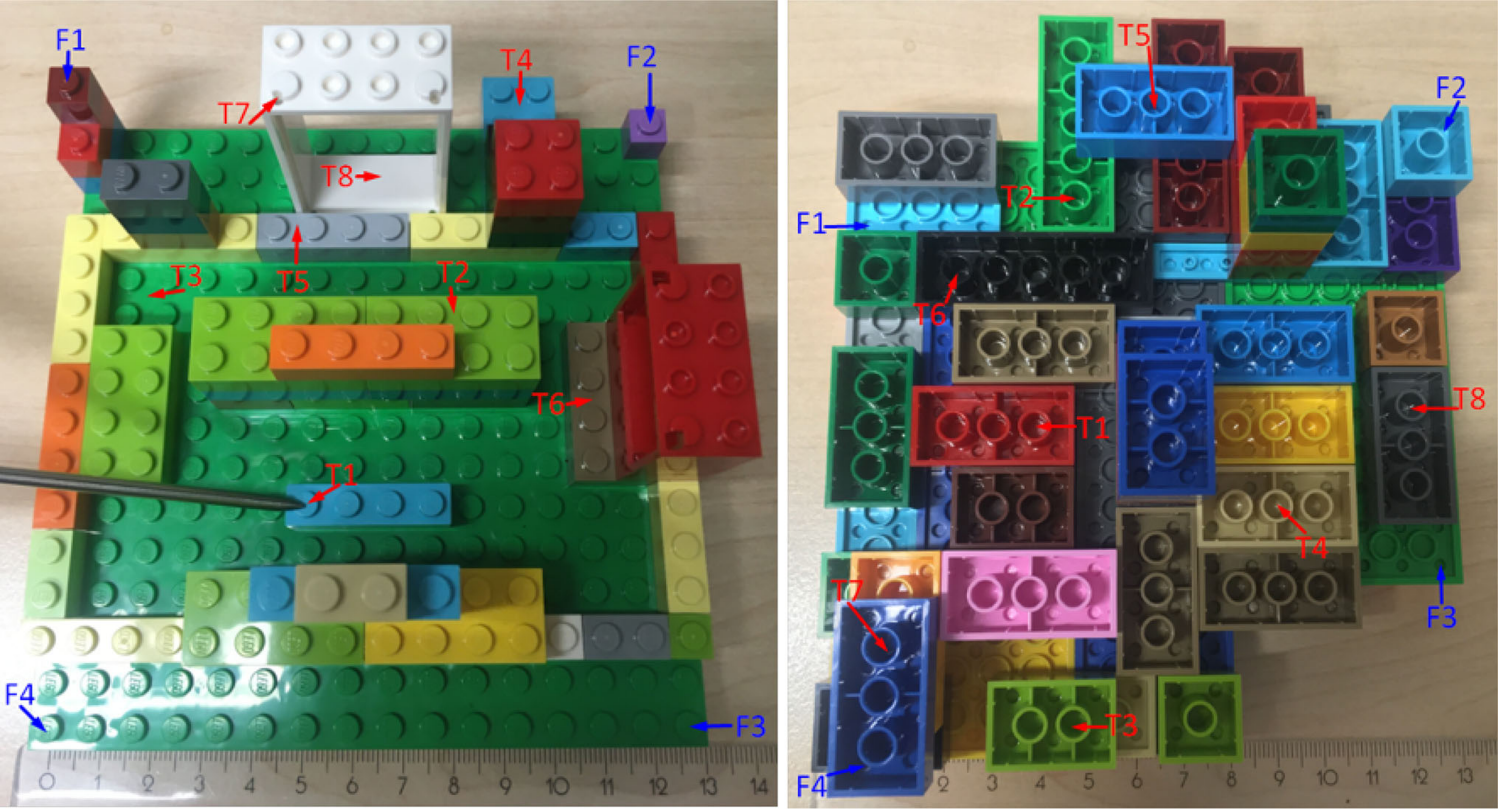
Left: The first Lego phantom with targets distributed on the plates of the Lego blocks. Right: The second phantom with targets inside the tubes of Lego blocks at the midpoint—not touching the plate. The targets are marked as T1, T2, etc. and four registration landmarks as F1, F2, F3 and F4

In this usability study, technical capabilities and human factors were evaluated by measuring participants’ performance on three system combinations as depicted in Fig. 3. First displays cross-sectional views on a screen and is termed as IGS in the analysis. Second projects visual guidance (VG) in the eyepieces of a surgical microscope (Leica M500 N, Leica Microscopy Systems, Heerbrugg, Switzerland). The third is a multi-modal combination of the first and the second (IGS+VG) and is displayed on a screen as well. These systems accept CT images of two custom-designed Lego phantoms (The Lego Group, Billund, Denmark), which are registered to the physical scene with four landmarks using a paired point-based registration [16]. The landmark combination that is non-collinear and non-coplanar was located on the phantom corners (see Fig. 4). In both phantoms, a 6D magnetic sensor acting as a dynamic reference frame (DRF) was glued on a Lego brick, which can be rigidly connected to the phantom.

**Fig. 5:**
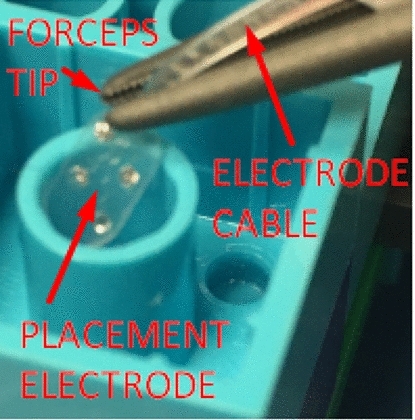
A participant placing a simulation brainstem electrode (5 mm *x* 3 mm) inside the Lego tube (5 mm in diameter) using a navigated forceps, without a direct view on the scene

Participants were asked to rely only on the systems in order to detect eight undisclosed and invisible targets, one at a time, randomly distributed for both phantoms. The targets were positioned at different depths and made challenging (e.g., behind the Lego wall). For the first phantom, the targets were selected on the Lego plates and identified using a surgical probe, while for the second phantom, they were selected inside the Lego tubes and identified using the forceps surgical instrument by placing a simulation electrode for ABI [17] inside the Lego tube target (Fig. 5).

**Fig. 6:**
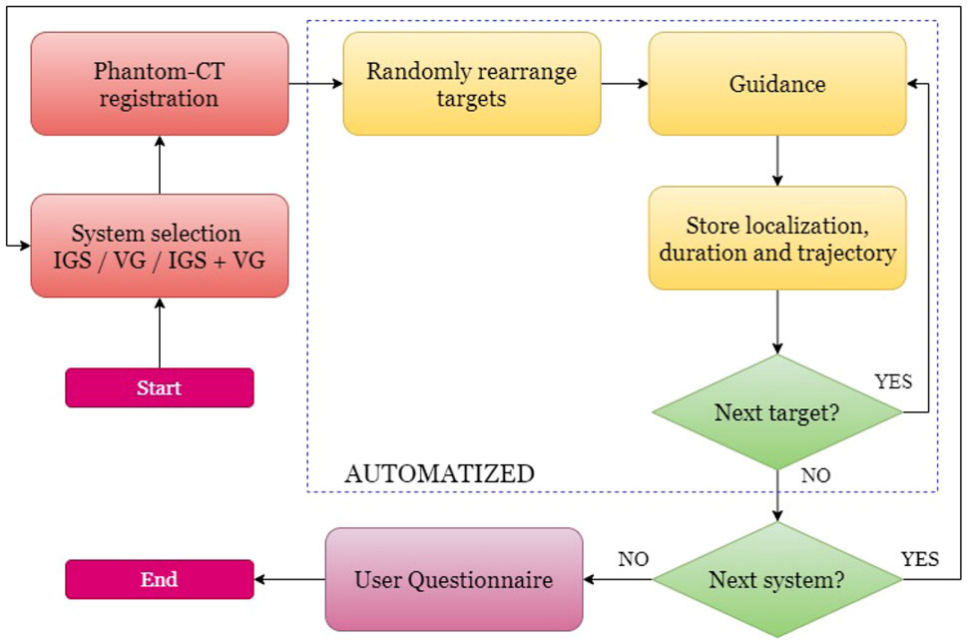
The usability experiment workflow

The experiment was semi-automatized with the workflow illustrated in Fig. 6. At the beginning, a phantom-to-CT rigid registration was performed with the fiducials being pinpointed on the Lego plates for each system using a surgical probe. The accuracy was inspected with several landmarks on the phantoms and if judged inaccurate, the registration was repeated. In order to avoid recalling same targets between the systems from short-term memory, the order is randomly rearranged in the code before the start. Following that, the participant was navigated after a beep sound. Once the target is localized, the position was stored with holding the surgical instrument for several seconds at the same location. The participant is updated with a beep sound that the target is stored and has a 5 s lapse to prepare for searching the next target from the common starting position. During the experiment, quantities such as trajectory, duration and position were automatically logged in a file. At the end of the experiment, the participant’s subjective opinion about the systems was evaluated by completing a user experience questionnaire.

Another supplementary video clip is available for a better overview of the study workflow.

### Quantitative evaluation

The Euclidian distance between the planned and user localized target, termed “user target error,” was measured on the Lego plates in the first phantom using the surgical probe. The hit or miss assessment was evaluated for placing the simulation brainstem electrode [17] in the Lego tubes in the second phantom using the forceps surgical instrument. Further, the average completion time and trajectory sum from the common starting point were evaluated for each accomplished target in both phantoms.

### Qualitative evaluation

**Fig. 7:**
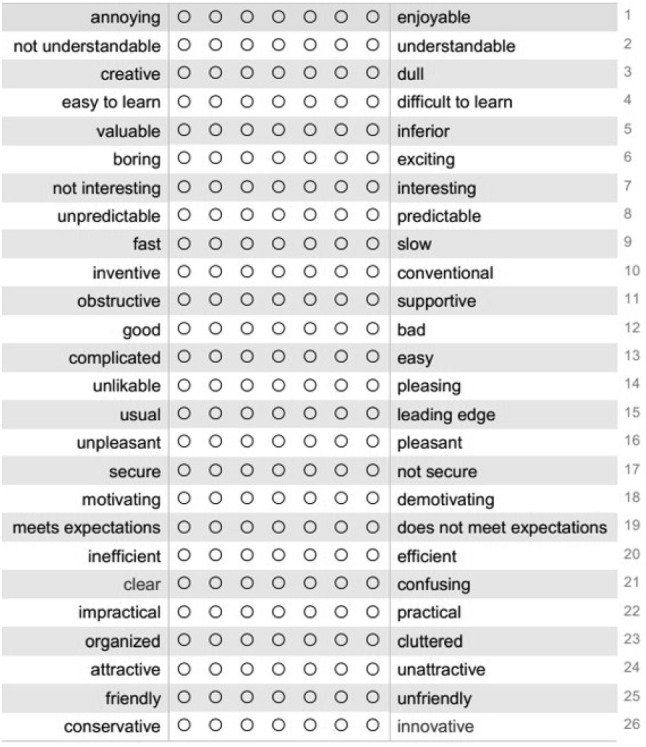
Sample of the UEQ presented to participants in the usability experiment obtained from www.ueq-online.org

A user experience questionnaire (UEQ) evaluates user’s subjective satisfaction and perceived workload with the systems. We use a UEQ questionnaire that is suggested as adequate for evaluating products with user interfaces [18]. This UEQ contains 26 items, where each item is presenting two terms with opposite meanings (e.g., attractive or unattractive, clear or confusing and conservative or innovative). The order of the terms (i.e., if the positive is left or right in an item) is randomized in the questionnaire to minimize answer tendencies [18]. The terms are scaled with 7 possible rankings for each item that the user has to select (e.g., from +3 for fully agree to 0 for neutral to $-3$ for fully disagree). Sample of the UEQ is shown in Fig. 7.

The items are classified into 6 scales: Attractiveness categorizes overall impression of the system; Perspicuity demonstrates how easy is to get familiar with the system; Efficiency categorizes interactive and practical impressions of the system; Dependability depicts if the system meets user expectations for the task at hand; Stimulation shows how exciting and motivating is the system for the task at hand; and Novelty outlines inventive and creative sides of the system.

## Results

In total, 14 participants included in the study were divided into two groups. The clinical experienced group consisted of 8 participants which are 4 ENT surgeons with IGS experience of several years; an IGS clinical intervention specialist with 25 years of experience; a radiology resident with 3 years of experience; a medical doctor that has been assisting in IGS interventions for 3 years; and a medical imaging clinical specialist with 25 years of experience. The non-clinical experienced group consisted of 6 participants who are 5 engineers ranging from 3 to 14 years of experience in the IGS field and a medical student doing his thesis in the medical imaging field.

Despite only 5-min introductory presentation about the experiment, the participants were able to learn by doing and with a quick learning curve. The average duration of the experiment per participant was 1 h and 15 min. The mean ± standard deviation FRE was $0.7 \pm 0.25$ mm. The TRE was not quantitatively evaluated, but it was visually verified with a probe on 2–3 trials if a registration was successful. During the experiment, the participants had no problems to localize targets in the images of the first phantom using the surgical probe as presented in Table 1. On the other hand, they were less successful and confident with the second phantom using surgical forceps with the actual 182 Lego tubes hit, 120 missed and 34 not evaluated/skipped from total 336 targets (all system combinations) as presented in Table 2. At the participant number 9, the 6D magnetic sensor on the forceps failed and the experiment continued with a surgical probe due to a missing backup sensor.

Following quantitative/qualitative analysis is performed in the R programming language.

### Quantitative analysis

A Wilcoxon signed rank test (two-sided, *p*-value $\le$ 0.01) confirmed the user target error (Table 1, *p*-value = 0.00015), trajectory (Table 2, *p*-value = 1.738e-05) and the duration time (Table 1, *p*-value = 1.3e-06 and Table 2, *p*-value = 5.481e-05) statistically significantly differ between the VG and IGS systems. This is a nonparametric test as the quantities were found to be not normally distributed (the boxplot distributions and Kolmogorov-Smirnov test, *p*-value $\le$ 0.05). The significance was also found between IGS+VG and IGS. The determined significance is identified for the calculated quantities in Tables 1 and 2.

**Fig. 8:**
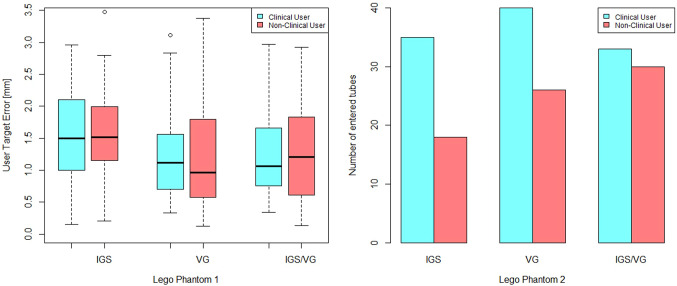
Left: Boxplots of the user target error for the first phantom. Right: Barplots of the achieved hit frequency for the second phantom

**Fig. 9:**
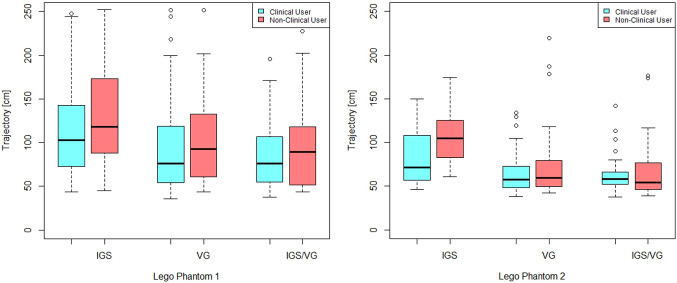
Boxplots of the trajectory sum for the first and the second phantom

**Fig. 10:**
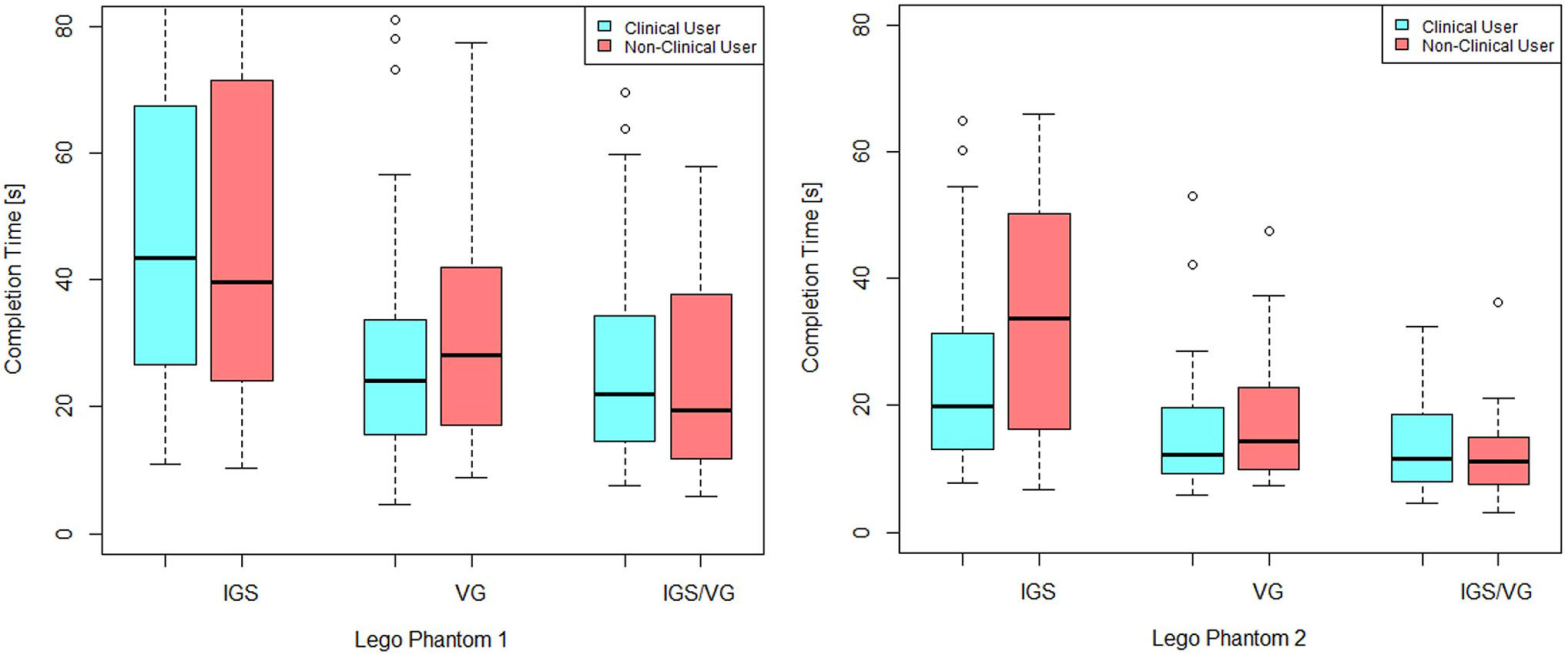
Boxplots of the duration time for the first and the second phantom

Further, Figs. 8, 9 and 10 show boxplots and barplots of the analyzed quantities for both phantoms categorized by the groups and systems.

**Table 1:** Experimentally determined mean ± standard deviation for user target error, trajectory and duration quantities for the first phantom

System	Error (mm)	Trajectory (cm)	Duration (s)
IGS	$1.60 \pm 0.82$	$134 \pm 98$	$59 \pm 54$
VG	$1.27 \pm 0.90^*$	$97 \pm 49$	$33 \pm 27^*$
IGS+VG	$1.31 \pm 0.81^*$	$89 \pm 43$	$28 \pm 21^*$

*The asterisk identifies the *p*-values that are significant at the 0.01 level

**Table 2:** Experimentally determined tube hit (missed) and mean ± standard deviation for trajectory and duration quantities for the second phantom

System	Hit (miss)	Trajectory [cm]	Duration [s]
IGS	53(48)	$94 \pm 42$	$29 \pm 19$
VG	66(34)	$70 \pm 35^*$	$16 \pm 10^*$
IGS+VG	63(38)	$65 \pm 28^*$	$13 \pm 6^*$

*The asterisk identifies the *p*-values that are significant at the 0.01 level

### Qualitative analysis

**Fig. 11:**
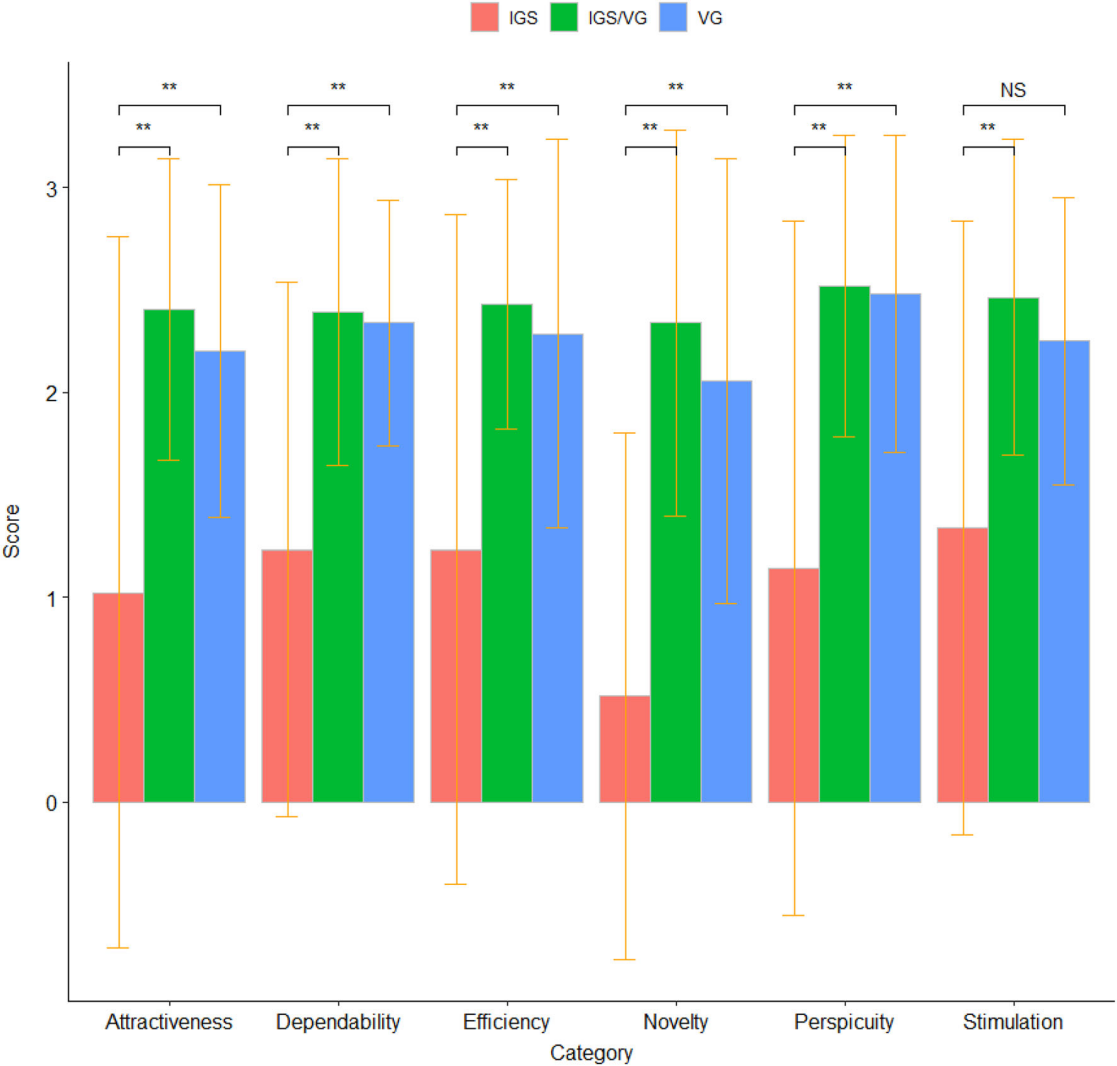
Barplot of the scale categories from the filled UEQ with the mean and standard deviation. The score range is from $-3.5$ to $+3.5$. **The double asterisk identifies the *p*-values that are significant at the 0.05 level

Figure 11 shows the analysis of subjective opinions of the participants from the UEQ. A *t* test with significance level 0.05 shows statistical difference between VG and IGS-only for categories Attractiveness (*p*-value = 0.033), Perspicuity (*p*-value = 0.015), Efficiency (*p*-value = 0.05), Dependability (*p*-value = 0.0097), and Novelty (*p*-value = 0.0021) and not for Stimulation (*p*-value = 0.0534). On the other hand, the statistical difference was not found between VG and IGS+VG (*t* test, significance level 0.05). Data did not differ between clinical and non-clinical experienced users (*t*-test, significance level 0.05).

## Discussion

As an alternative to the current state of the art, we presented a new visual interface for simultaneous surgical targeting by visualizing the 3D positioning data of tracked interventional instruments with a few easy-to-learn sensory cues. At design time, the authors were particularly inspired by heads-up displays in aviation industry in order to visualize surgical orientation intuitively, responsively and with minimum possible information. We aimed to improve surgical experience in specialized targeting interventions with an adequate, highly specialized and minimalistic interface that does not require frequent interruptions during intervention to look on a screen for navigation, especially because the studies shown that this causes additional cognitive load for surgeons and therefore can limit use of surgical navigation [9]. In addition, our clinical experience as well as discussions with surgeons advised us to avoid showing numbered distances, but rather to encode them visually. Having said that, our interface is designed to be as simple as possible and guide surgeon toward the target point with a few instinctive visual objects that can be displayed for minimum mental load and distractions to site.

Though it was not considered here, if required anatomical landmarks of interest or critical structures marked in preoperative images can be displayed relative to the target point for improved guidance (see the supplementary video).

The first results were obtained in a usability study that served as a primary means of demonstrating if the system can be used efficiently and as intended. Multidisciplinary team of several clinical and non-clinical experts participated in the study. The presented system is compared independently and in a combination with cross-sectional views navigation with two reoccurring tasks designed to localize targets in images of phantoms. The phantom scenes were covered in order to evaluate user interface capabilities without relying on the scene feedback. The obtained quantitative results demonstrated that both clinical and non-clinical experts were able to localize targets in the phantom image with millimetric precision using all three system combinations, and with the first group achieving shorter trajectories. The mean accuracy is within a low millimetric margin, suggesting to be optimal, considering that typical neurosurgical procedures are in this accuracy range [1].

Conversely and unexpectedly, a high miss rate was observed when guiding an instrument into a Lego tube. It is surprising since the users could additionally rely on the tactile sensation provided against the tube borders. Initially, we designed this experiment as opposite to the first one, where the target destination was set on the “open” surface of Lego bricks, in order to obtain a preliminary insight into a possibility of deploying brainstem electrodes to a marked region. The electrode positioning was not critical in this task; however, it should be noted that this is one of the main drivers of auditory performances in ABI procedures [19]. Several factors could contribute to more likely miss the target: primarily the scene invisibility; navigation error originating from EMT and image registration (though the accuracy was visually inspected after each image registration); users terminating too fast or absence of verification biases; and visualization conditioning users to overlay two visual objects instead of the complete end-to-end trajectory guidance. This signalizes that we should not take our results with the first phantom for granted and that improvements would be required for certain clinical scenarios. Indeed, to accommodate the interface for deep brain stimulation, it would be necessary to consider designing and following a safe trajectory that avoids intersecting vital structures and maximizes the likelihood of hitting the target.

The qualitative analysis showed that the participants perceived the proposed visual guidance (also in combination with cross-sectional views) highly positive in all UEQ categories, and particularly in Novelty scale. The cross-sectional views (stand-alone solution) were significantly outperformed both in quantitative and qualitative evaluations, which was expected considering that in general they are not specialized and optimized for target guidance.

It is interesting, however, that coupling two interfaces demonstrated high usability results with high localization accuracy and small trajectory and duration time, which indicates potential for possible improvements. A similar conclusion was reached in [20] when combining audiovisual display for an ablation needle placement. Generally, neuropsychologists show that by accumulating the amount of information with good quality will help observers make better decisions about the target especially in case of low prevalence (the target can be located anywhere in the phantoms) [21].

We demonstrated the visualization concept on a tracked surgical probe and forceps typically used in clinical interventions. Although EMT is explored in this work, there are no restrictions for OT. The visualization is independent from the image registration method. However, it would be informative for a future clinical scenario in order to consider optimal TRE. Therefore, visualizing predicted TRE uncertainty as suggested in this work is beneficial for surgeons to be aware of potential misalignments and react accordingly to minimize errors as much as possible. In addition, optimal placements of the DRF shall also be considered since the distance from the instrument tip and the target point will impact the final accuracy [15].

While the merits of this study indicate high usability-related aspects of the projected visualization in the oculars of a surgical microscope, it will not reflect live experience of surgeons during the procedure. In order to further strengthen applicability of the proposed system, we are currently evaluating a prototype version for auditory brainstem implantation in a preclinical setting with a human head specimen. This study will consider the compatibility issues of EMT with the surrounding surgical scene (e.g., other instruments/devices/tables), exact registration approach/accuracy [6], intraoperative brain shift deformation and less rigid and sharp targets than Lego bricks. The preliminary feedback from neurosurgeons indicate that the interface is potentially helpful and useful during surgery, with its main strength to be displayed directly in the microscope without making the scene too cluttered. Due to the complexity of work, it will be considered in a separate paper.

## Conclusion

This work highlights concepts of a novel visual display intended to simplify and foster the current state of the art in intraoperative surgical guidance. Our system focuses on ease of use and an optimal surgical experience during target localization. The interface seamlessly integrates into the surgical reality and fits existing software environments (source code is available upon request). We designed a usability study with Lego phantoms, which can be easily customized compared to the standard skull base models, to rapidly assess and improve HCI in visual or auditory displays. The results from the proposed study imply high usability-related aspects of the system, currently limited to phantom experiments. Therefore, upcoming evaluations will be performed under preclinical in-situ condition and for auditory brainstem implantation.

## Supplementary Information

Below is the link to the electronic supplementary material.

Supplementary material 1 (mp4 226927 KB)

Supplementary material 2 (mp4 198114 KB)
